# Inhibition of monogalactosyldiacylglycerol synthesis by down-regulation of MGD1 leads to membrane lipid remodeling and enhanced triacylglycerol biosynthesis in *Chlamydomonas reinhardtii*

**DOI:** 10.1186/s13068-022-02187-x

**Published:** 2022-08-27

**Authors:** Jun-Woo Lee, Min-Woo Lee, Chun-Zhi Jin, Hee-Mock Oh, EonSeon Jin, Hyung-Gwan Lee

**Affiliations:** 1 Cell Factory Research Center, Korea Research Institute of Bioscience and Biotechnology (KRIBB), Daejeon, 34141 Republic of Korea; 2 Department of Life Science, Hanyang University, Seoul, 04763 Republic of Korea; 3 LMO Team, National Institute of Ecology, Seocheon, 33657 Republic of Korea; 4 Department of Environmental Biotechnology, University of Science & Technology (UST), Daejeon, 34113 Republic of Korea

**Keywords:** Membrane lipid remodeling, Monogalactosyldiacylglycerol, *Chlamydomonas reinhardtii*, Triacylglycerol, Microalgal biodiesel

## Abstract

**Background:**

Membrane lipid remodeling involves regulating the physiochemical modification of cellular membranes against abiotic stress or senescence, and it could be a trigger to increase neutral lipid content. In algae and higher plants, monogalactosyldiacylglycerol (MGDG) constitutes the highest proportion of total membrane lipids and is highly reduced as part of the membrane lipid remodeling response under several abiotic stresses. However, genetic regulation of MGDG synthesis and its influence on lipid synthesis has not been studied in microalgae. For development of an industrial microalgae strain showing high accumulation of triacylglycerol (TAG) by promoting membrane lipid remodeling, MGDG synthase 1 (*MGD1*) down-regulated mutant of *Chlamydomonas reinhardtii* (*Cr-mgd1*) was generated and evaluated for its suitability for biodiesel feedstock.

**Results:**

The *Cr-mgd1* showed a 65% decrease in *CrMGD1* gene expression level, 22% reduction in MGDG content, and 1.39 and 5.40 times increase in diacylglyceryltrimethylhomoserines (DGTS) and TAG, respectively. The expression levels of most genes related to the decomposition of MGDG (plastid galactoglycerolipid degradation1) and TAG metabolism (diacylglycerol *O*-acyltransferase1, phospholipid:diacylglycerol acyltransferase, and major lipid droplet protein) were increased. The imbalance of DGDG/MGDG ratio in *Cr-mgd1* caused reduced photosynthetic electron transport, resulting in less light energy utilization and increased reactive oxygen species levels. In addition, endoplasmic reticulum stress was induced by increased DGTS levels. Thus, accelerated TAG accumulation in *Cr-mgd1* was stimulated by increased cellular stress as well as lipid remodeling. Under high light (HL) intensity (400 µmol photons/m^2^/s), TAG productivity in *Cr-mgd1–HL* (1.99 mg/L/d) was 2.71 times higher than that in wild type (WT–HL). Moreover, under both nitrogen starvation and high light intensity, the lipid (124.55 mg/L/d), TAG (20.03 mg/L/d), and maximum neutral lipid (56.13 mg/L/d) productivity were the highest.

**Conclusions:**

By inducing lipid remodeling through the *mgd1* gene expression regulation, the mutant not only showed high neutral lipid content but also reached the maximum neutral lipid productivity through cultivation under high light and nitrogen starvation conditions, thereby possessing improved biomass properties that are the most suitable for high quality biodiesel production. Thus, this mutant may help understand the role of *MGD1* in lipid synthesis in *Chlamydomonas* and may be used to produce high amounts of TAG.

**Supplementary Information:**

The online version contains supplementary material available at 10.1186/s13068-022-02187-x.

## Background

Microalgal lipids consist of galactolipids, phospholipids, and neutral lipids that are widely used in biodiesel production, cosmetics, and pharmaceutical industries. In microalgal biodiesel production, neutral lipids are practically more preferred as biodiesel feedstock than galactolipids and phospholipids due to lack of sulfur and phosphorous in them [1]. The sulfur present in thylakoid membrane lipids, especially sulfoquinovosyldiacylglycerol, has detrimental effects on the quality of biodiesel, and the phosphorous in phospholipids disturbs the transesterification reaction, thus leading to low yield of biodiesel [2]. Moreover, one of the important parameters in microalgal biodiesel production is the fatty acid composition, which could affect fluidity at low temperature and oxidative stability of biodiesel [3]. Low concentration of long-chain saturated fatty acids is beneficial to maintain fluidity at low temperature, and high concentration of saturated fatty acids (SFAs) and monounsaturated fatty acids (MUFAs) is suitable for oxidative stability [4]. Thus, artificial regulation of MGDG levels could induce membrane lipid remodeling, and therefore, research on the regulation of compositions of lipids and fatty acids in microalgae have been widely conducted for production of high quality biodiesel.

Membrane lipids, which are formed as a bilayer, form the containment unit of cells and micro-organelles and play functional roles, such as in the biosynthesis of biomolecules, regulation of cell growth, and control of the biophysical homeostasis. Among membrane lipids, monogalactosyldiacylglycerol (MGDG) in higher plants and algae accounts for more than 50% of the total membrane lipids, and it plays an important role in the formation and maintenance of chloroplasts and thylakoid membranes [5, 6]. In *Arabidopsis thaliana*, MGDG is synthesized by two types of MGDG synthases—*AtMGD1* (type A) and *AtMGD2* and *AtMGD3* (type B) [7, 8]. Both knock-down and knock-out mutants of *AtMGD1* showed impaired thylakoid membrane energization and decreased photoprotective capacity or the complete dysfunction of photosynthetic activities. Aronsson et al. (2008) confirmed that MGDG deficient mutants, developed by down-regulation of *AtMGD1,* showed inhibition of the xanthophyll cycle function and significantly reduced heat dissipation ability in response to excess light [9]. In contrast, *AtMGD2* and *AtMGD3* are reported to only contribute to galactolipid synthesis under phosphate-deficient conditions. In *Chlamydomonas reinhardtii,* only type A MGDG synthase was identified, and its gene is named *CrMGD1* (Cre13.g585301.t1.1) [10]; however, the relationship between the regulation of expression level of *CrMGD1* and its influence on lipid synthesis has not been studied.

Higher plants and microalgae show membrane lipid remodeling, which is one of the most effective acclimation responses against environmental challenges or abiotic stresses, such as drought [11], salt [12], chilling [13], heat [14], and nutritional deficiency [15]. The structural modification of cellular membrane lipids is flexibly regulated by lipid metabolism, membrane repair response, fatty acid trafficking, cellular signaling, and homeostasis [16, 17]. Increased cellular levels of diacylglycerol (DAG) pools and acyl-CoA, which are derived from membrane lipid remodeling by lipases, desaturases, and acyltransferases, are incorporated into triacylglycerol (TAG) biosynthesis, resulting in the accumulation of lipid droplets. Modification of galactolipids, which are dominant in the membrane and show most fluctuations in response to abiotic stresses, is closely correlated with the biosynthesis of signaling compounds for stress response and TAG metabolism. In *Chlamydomonas*, MGDG-specific lipase PGD1 (plastid galactoglycerolipid degradation1) was shown to play a critical role in shifting the fatty acid flux from MGDG to TAG and enhancing thylakoid membrane stability in response to nitrogen starvation [18, 19]. Thus, artificial regulation of MGDG levels could induce membrane lipid remodeling, resulting in TAG accumulation and enhanced stress resistance.

In this study, *Cr-mgd1* mutant with down-regulated *CrMGD1* was generated to investigate the induction of membrane lipid remodeling and its effect on TAG metabolism. Molecular and physiological analyses were performed to explain the effect of MGDG reduction on lipid remodeling, cellular response, and TAG metabolism. Based on the physiological analysis of *Cr-mgd1*, reduction of MGDG levels also caused a decrease in photosynthetic activity and induction of cellular stress. Considering decreased light utilization of *Cr-mgd1*, biomass, lipid, and TAG productivity were investigated under moderate and high light intensities.

## Results and discussion

### Promotion of membrane lipid remodeling by inhibition of MGDG synthesis

**Fig. 1:**
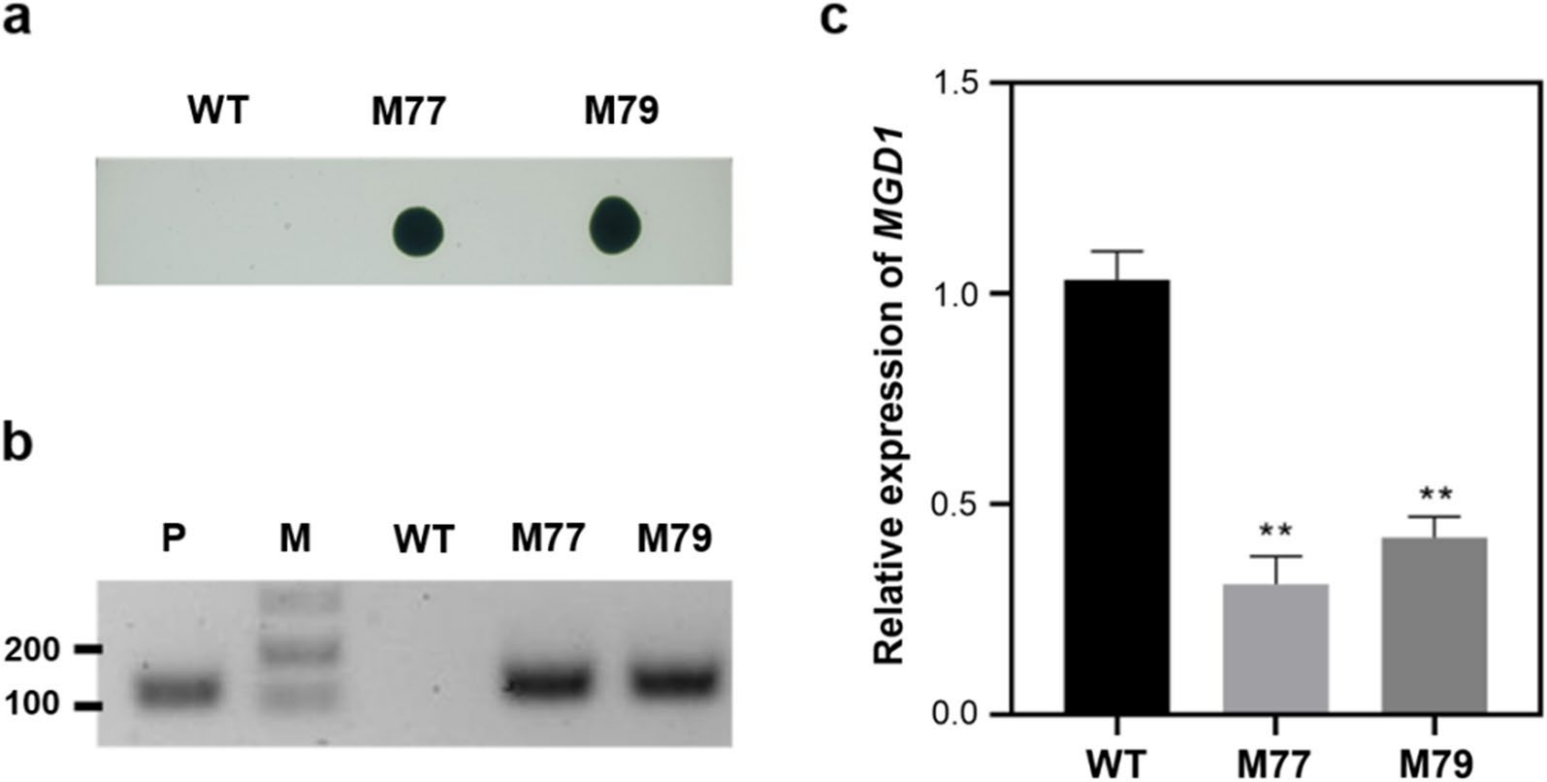
Confirmation of *Cr-mgd1* knockdown strains. **a** Paromomycin susceptivity. **b** *aphVIII* amplification in cDNA. **c** Relative gene expression of *CrMGD1* by qRT-PCR. P: pCrMGD1 vector; M: Marker; WT: wild-type. Relative expression levels were calculated by normalizing the values to those of *Chlamydmonas β-subunit-like polypeptide* (*CBLP*). Significant differences between WT and *Cr-mgd1* are represented with asterisks based on Holm–Sidak method (**P < 0.01). The error bars represent the standard deviation (SD)

**Fig. 2:**
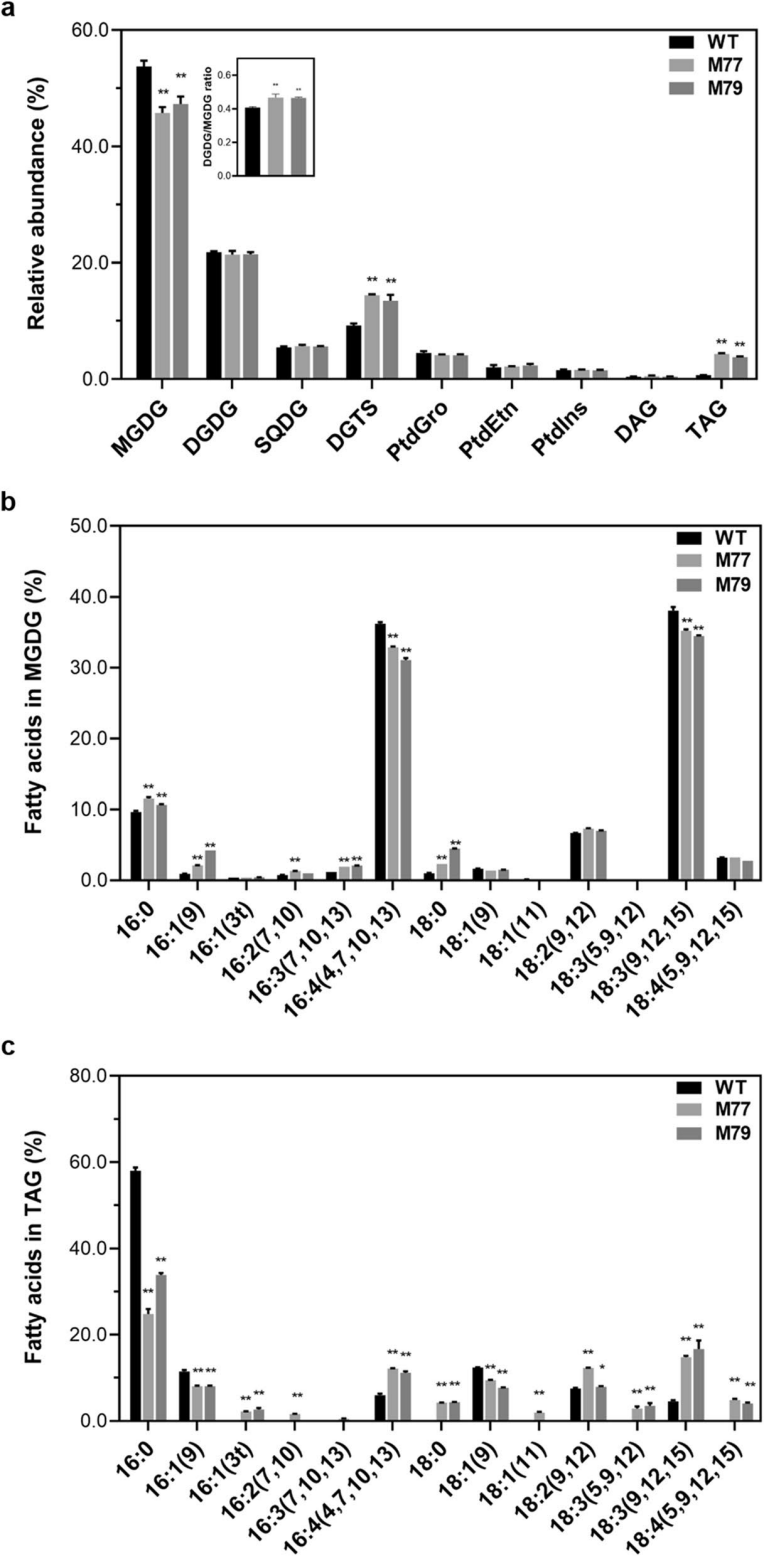
Membrane lipids and fatty acids composition in the WT and *Cr-mgd1*. **a** Quantitative analysis of lipid classes. **b** Fatty acid profiling of MGDG under moderate-light conditions. **c** Fatty acid profiling of TAG under moderate-light conditions. Profiling of lipids and fatty acids was performed using GC–FID and was normalized to dry cell weight. Significant differences between WT and *Cr-mgd1* are represented with asterisks based on Holm–Sidak method (**P < 0.01), and the error bars represent the standard deviation (SD). WT: wild-type; MGDG: monogalactosyldiacylglycerol; DGDG: digalactosyldiacylglycerol; SQDG: sulfoquinovosyldiacylglycerol; DGTS: digalactosyl-*N*,*N*,*N*-trimethylhomoserine; PtdGro: phosphatidylglycerol; PtdEtn: phosphatidylethanolamine; PtdIns: phosphatidylinositol; DAG: diacylglycerol; TAG: triacylglycerol

*CrMGD1* (Cre13.g585301) down-regulated mutants of *C. reinhardtii* CC-124 were successfully generated using artificial miRNA-mediated method and confirmed using genotypic and phenotypic methods. *Cr-mgd1*mutatns, M77, and M79, which were resistant to 50 µg/mL paromomycin, were validated to have the partial amplification of the paromomycin resistance gene (*aphVIII*) (Fig. 1a, b). Southern blotting analysis confirmed that one copy of the *aphVIII* gene was integrated into each mutant’s gDNA (Additional file 1). qRT-PCR analysis revealed that the expression levels of *CrMGD1* gene in M77 and M79 were down-regulated by 70% and 59%, respectively (Fig. 1c). Based on lipid profiling analysis, the reduction in MGDG content in *Cr-mgd1* due to downregulation of *CrMGD1* induced lipid remodeling, such as increased diacylglyceryltrimethylhomoserines (DGTS) and TAG content (Fig. 2a). While MGDG content in *Cr-mgd1* was significantly reduced by 22%, DGTS and TAG were particularly increased 1.39 and 5.40 times, respectively.

Fatty acid profiling of MGDG in *Cr-mgd1* revealed that C16:4(4,7,10,13) and C18:3(9,1215), which are the major fatty acids in MGDG, were reduced, whereas C16:1(9), C16:3(7,10,13), and C18:0 were increased (Fig. 2b). Zauner et al. identified MGDG-specific Δ4-desaturase from *Chlamydomonas* (*CrΔ4FAD*) and confirmed that its overproduction not only increased C16:4(4,7,10,13) content but also specifically increased the MGDG content [20]. They suggested that *CrΔ4FAD* was linked to MGDG and has a specific function regarding MGDG. In addition, Yang et al. reported that *CrΔ4FAD* and fatty acid desaturase 6 from *Chlamydomonas* (*CrFAD6*) contributed to the stabilization of MGDG [21]. To analyze the relationship between *CrMGD1* and desaturases, expression levels of *CrFAD6* (Cre13.g590500) and *CrΔ4FAD* (Cre01.g037700) were investigated by qRT-PCR. The results revealed that the expression of both desaturase genes in *Cr-mgd1* was down-regulated compared to WT (Additional file 2). Therefore, the reduction in MGDG content in *Cr-mgd1* was also due to the reduction in fatty acid desaturase levels required for MGDG maturation.

The amount of TAG in *Cr-mgd1* mutant was 5.4-fold higher compared to that in WT (Fig. 2a), and fatty acid composition of TAG was enriched in *Cr-mgd1* mutant (Fig. 2c). A decrease in C16:4(4,7,10,13) and C18:3(9,12,15) was observed in total fatty acid composition (Additional file 3). The immature MGDG exported fatty acids, and they were either incorporated into TAG or used for the maturation of MGDG by desaturase enzymes. The down-regulated expression levels of *FAD6* and *CrΔ4FAD* and relative abundance of digalactosyldiacylglycerol (DGDG) maintained in *Cr-mgd1* mutant suggested that the exported fatty acids from immature MGDG were preferably integrated into TAG rather than being used in the synthesis of MGDG and DGDG. Moreover, DGDG/MGDG ratio increased up to 14% in *Cr-mgd1* due to decreased MGDG content and not due to changes in the DGDG content (Fig. 2a).

The DGDG/MGDG ratio is an important factor that determines the shape and stability of the chloroplast membrane and participates in stress response and tolerance [22]. Under various stresses, such as freezing, drought, and high salt conditions, DGDG/MGDG ratio increases to adapt to these stresses and maintains the chloroplast membranes in a bilayer conformation. In addition, the imbalance of DGDG/MGDG ratio contributes to the accumulation of TAGs and induces a stress response. The ferredoxin-5 (*FDX5*) null mutant of *C. reinhardtii* showed changes in the DGDG/MGDG ratio and increased TAG content [21]. Therefore, imbalance of DGDG/MGDG ratio in *Cr-mgd1* might be associated with lipid remodeling causing TAG accumulation and induction of protective mechanisms to overcome stress.

### Molecular and physiological analysis of *Cr-mgd1,* and its contribution to TAG biosynthesis

**Fig. 3:**
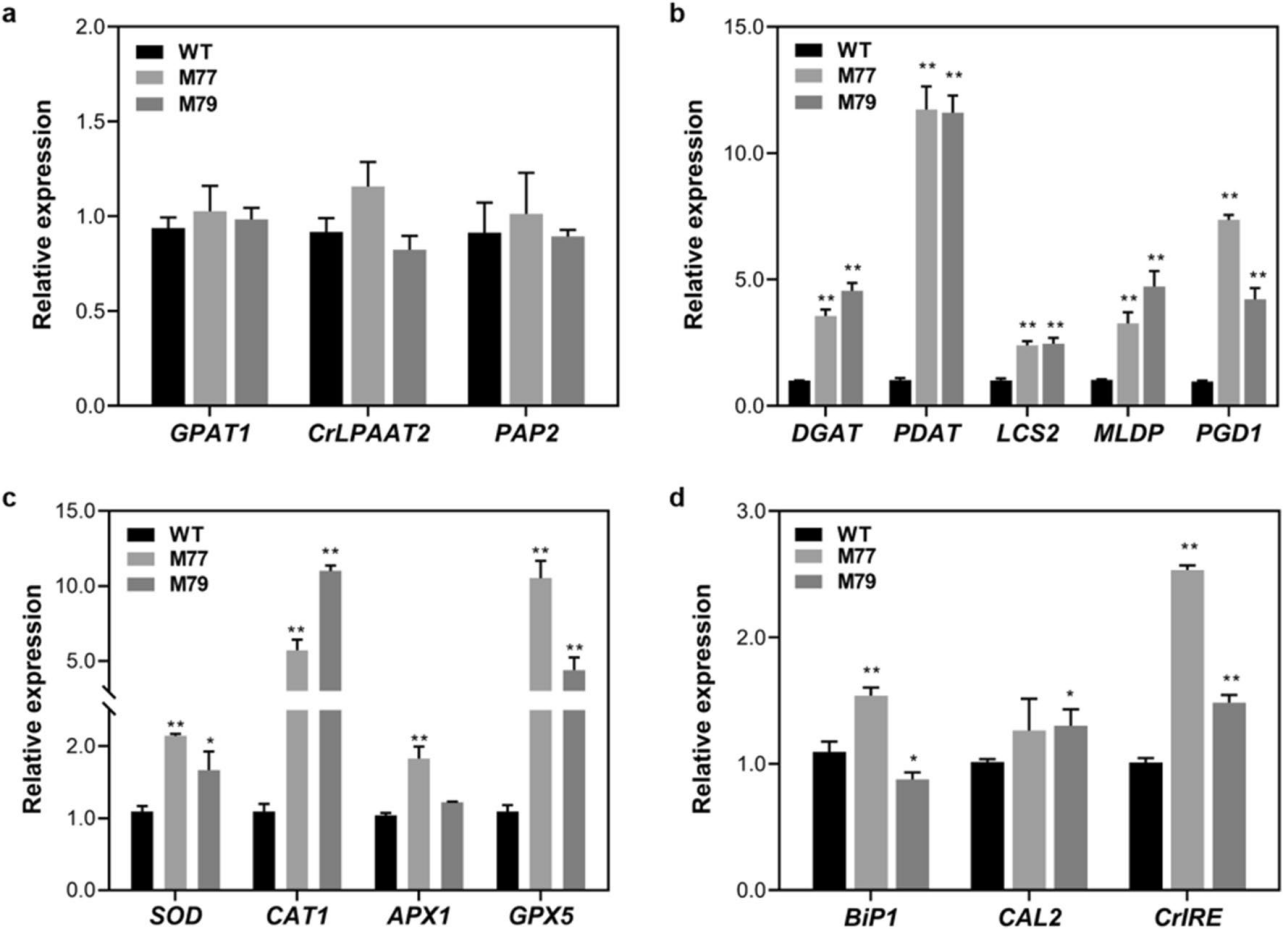
Relative gene expression analysis using quantitative real-time PCR. **a** DAG biosynthesis. **b** TAG biosynthesis. **c** ROS scavenging. **d** ER stress marker. Relative expression levels were calculated by normalizing the values to those of *Chlamydmonas* β-subunit-like polypeptide (*CBLP*). Significant differences between WT and *Cr-mgd1* are represented with asterisks based on Holm–Sidak method (**P < 0.01), and the error bars represent the standard deviation (SD). WT: wide-type; DGAT1: diacylglycerol *O*-acyltransferase1; PDAT: phospholipid:diacylglycerol acyltransferase; LCS2: long-chain acyl-CoA synthetase2; MLDP: major lipid droplet protein; PGD1: plastid galactiglycerolipid degradation1; SOD: superoxide dismutase; CAT1: catalase1; APX1: ascorbate peroxidase1; GPX5: glutathione peroxidase5; GPAT1: glycerol 3-phosphate; CrLPAAT2: 2‐lysophosphatidic acid acyltransferase2 from *Chlamydomonas reinhardtii*; PAP2: phosphatidic acid phosphatase2; BiP1: binding protein1; CAL2: calreticulin2; CrIRE: *Chlamydomonas reinhardtii* inositol-requiring enzyme1

To identify the mechanism of lipid remodeling induction by inhibition of MGDG synthesis, several important genes related to the TAG synthesis pathway and membrane lipid degradation were investigated. The expression levels of glycerol 3-phosphate (*GPAT1,* Cre06.g273250.t1.2), *Chlamydomonas reinhardtii* 2‐lysophosphatidic acid acyltransferase2 (*CrLPAAT2,* Cre17.g738350.t1.2), and phosphatidic acid phosphatase2 (*PAP2,* Cre05.g240000.t1.2) which catalyze the reactions involved in DAG synthesis from G3P, were not changed (Fig. 3a). However, the expression levels of diacylglycerol O-acyltransferase1 (*DGAT1,* Cre01.g045903.t1.1) and phospholipid:diacylglycerol acyltransferase (*PDAT,* Cre02.g106400.t1.1), which are the key transferases of fatty acids to DAGs, were up-regulated (Fig. 3b). Interestingly, long-chain acyl-CoA synthetase2 (*LCS2,* Cre13.g566650.t1.2) and *PGD1* (Cre03.g193500.t1.2), which participate in free-fatty acid metabolism, and major lipid droplet protein (*MLDP,* Cre09.g405500.t1.1), which is usually accompanied with increased lipid droplets, were also up-regulated. Based on gene expression analysis, the regulation of MGDG synthesis seemed to widely influence the lipid metabolism, and the hypothetical mechanism of the increase in TAG content in *Cr-mgd1* was deduced as follows—inhibition of MGDG synthesis initially contributed to the increased DAG pool and free fatty acids in the chloroplast and cytosol (Additional file 4), which were not used to synthesize MGDG and were released by the decomposition of MGDG by *PGD1* or catalysis by *LCS2*. Thus, activation of *DGAT1* and *PDAT* might have a synergistic effect on TAG accumulation.

**Fig. 4:**
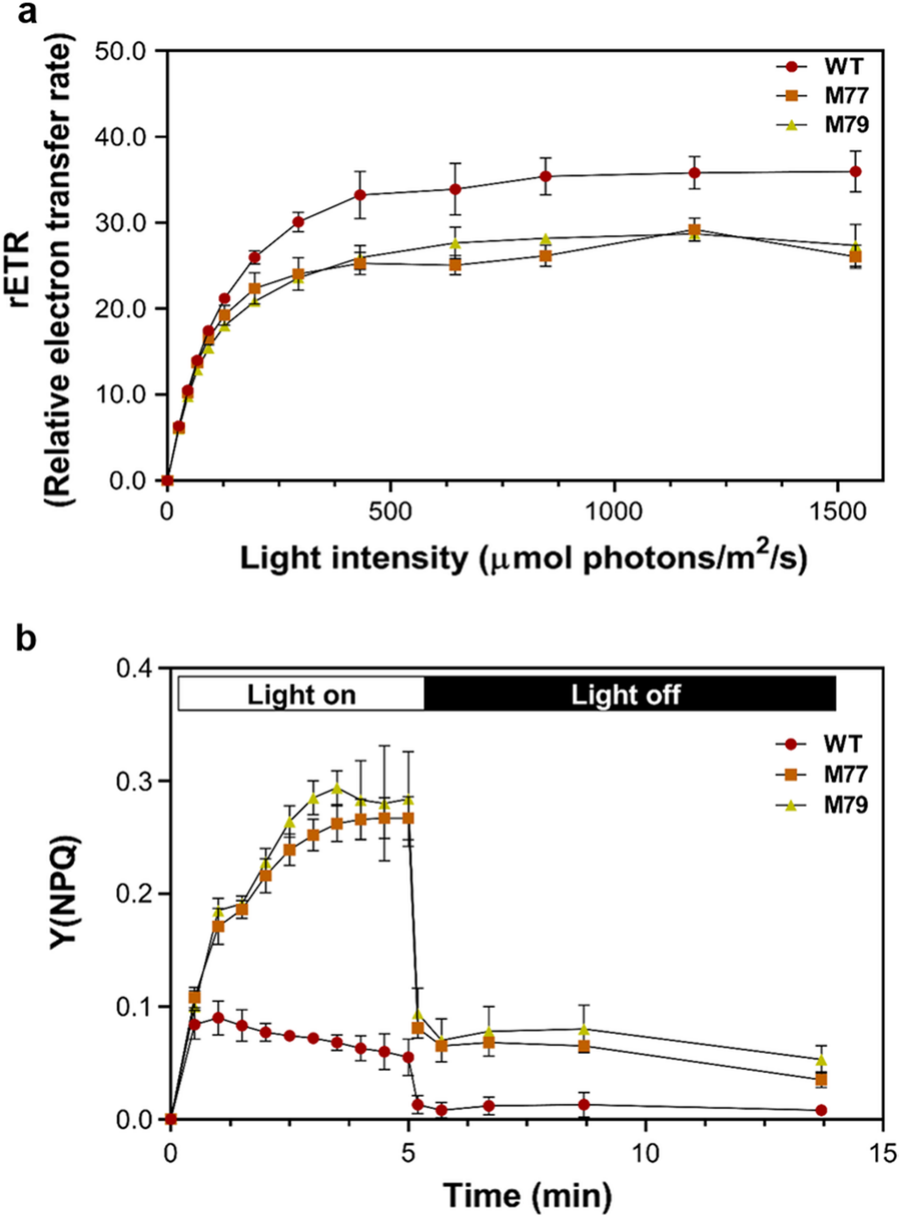
Measurement of photosynthetic activity using pulse amplitude modulation. **a** rETR. **b** Y(NPQ). Y(NPQ) was measured depending on time period under 1500 µmol photons/m^2^/s, and rETR was determined as a light response curve and measured as described in “Methods”. The error bars represent the standard deviation (SD)

Unstable chloroplast membrane due to the imbalance of DGDG/MGDG ratio in *Cr-mgd1* might cause reduction in the photosynthetic electron transport, resulting in a low relative electron transfer rate (rETR) and Y(NPQ) (Fig. 4a, b). While the rETR level was similar till 100 µmol photons/m^2^/s, the difference in the levels gradually increased with the increase in light intensity. A decrease in the maximum rETR level implied that the photosynthetic electron transport and light utilization for absorbance and/or dissipation under high light intensity were reduced [23]. In addition, an abnormal Y level in non-photochemical quenching (NPQ) indicated that non-photochemical energy dissipation pathways were more activated than the absorption of light energy pathways. Excess energy that could not be utilized in photosynthesis in *Cr-mgd1* might generate reactive oxygen species (ROS), which could increase the photo-oxidative damage. Increased levels of ROS (Additional file 5) and scavenging enzymes—superoxide dismutase (*SOD,* Cre02.g096150.t1.2), catalase1 (*CAT1,* Cre09.g417150.t1.2), ascorbate peroxidase1 (*APX1,* Cre02.g087700.t1.2), and glutathione peroxidase5 (*GPX5,* Cre10.g458450.t1.1)—were observed in *Cr-mgd1* (Fig. 3c). Photosynthetic efficiency and photoprotection were affected when photosynthetic organisms were exposed to abiotic stresses, such as high light intensity or chemical treatment [24]. Unstable chloroplast membrane due to *CrMGD1* knockdown might contribute to the increased TAG synthesis by causing less light energy utilization and increased ROS levels.

**Fig. 5:**
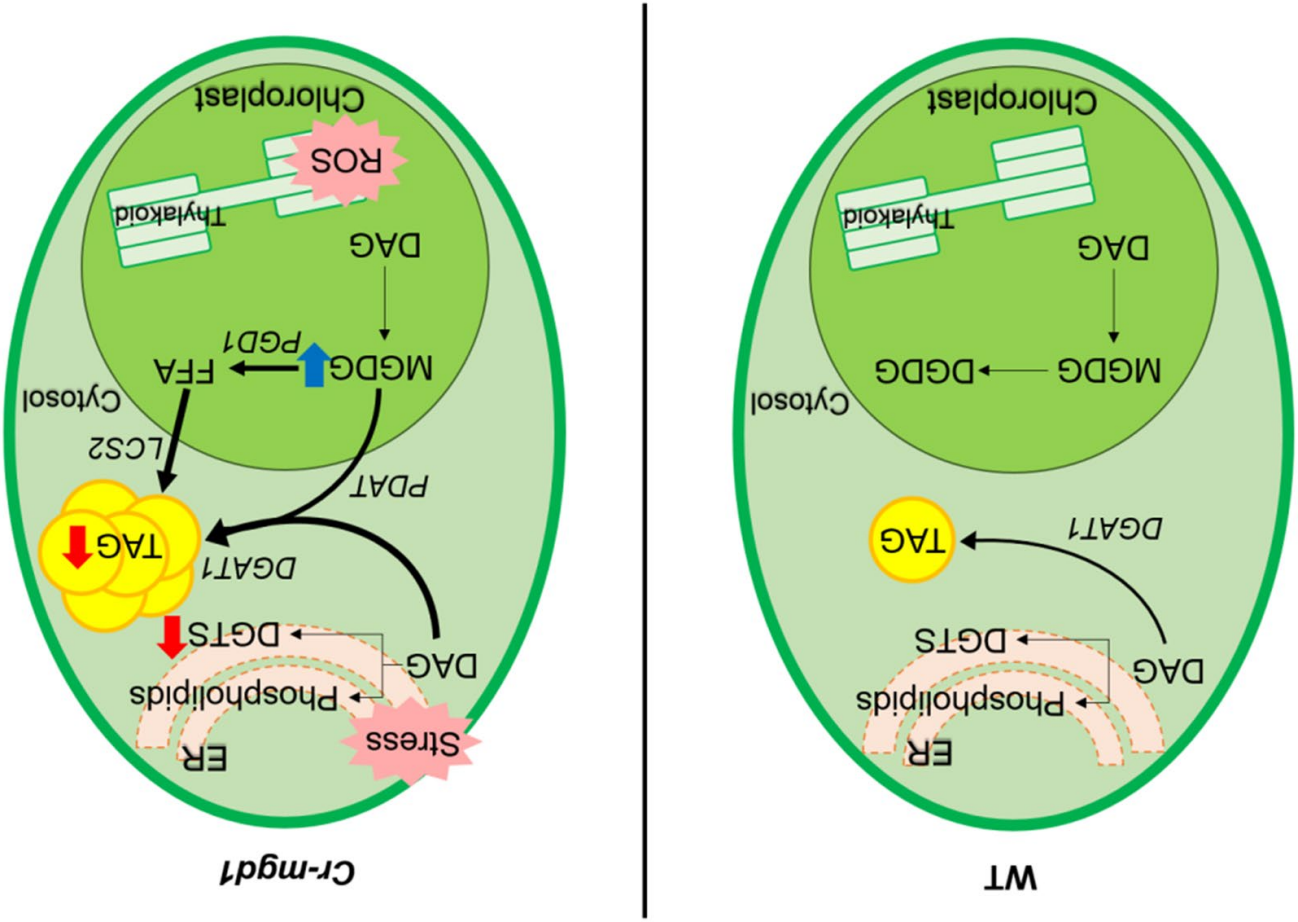
Hypothetical lipid synthesis pathway of *Cr-mgd1*. DGAT1: diacylglycerol *O*-acyltransferase1; PDAT: phospholipid:diacylglycerol acyltransferase; LCS2: long-chain acyl-CoA synthetase2; PGD1: plastid galactiglycerolipid degradation1; MGDG: monogalactosyldiacylglycerol; DGDG: digalactosyldiacylglycerol; DGTS: digalactosyl-*N*,*N*,*N*-trimethylhomoserine; DAG: diacylglycerol; TAG: triacylglycerol

Interestingly, the increased DGTS in *Cr-mgd1* might induce endoplasmic reticulum (ER) stress (Fig. 2a). The expression levels of binding protein1 (*BiP1*, Cre02.g080700.t1.2), inositol-requiring enzyme1 from *Chlamydomonas reinhardtii* (*CrIRE*, Cre08.g371052.t1.1), and calreticulin2 (*CAL2*, Cre01.g038400.t1.2), which are specifically activated in response to ER stress, were relatively up-regulated in *Cr-mgd1* (Fig. 3d). In *Chlamydomonas*, the change in DGTS content is considered one of the most important factors in the induction of ER stress and TAG accumulation [25]. The increase in DGTS content, which occurred due to tunicamycin (ER stress inducer) treatment, induced ER stress and coincided with increased TAG content, but decreased MGDG content [26]. Conversely, the decrease in DGTS content due to down-regulation of *BTA1* also induced ER stress and coincided with similar lipid remodeling patterns, that is, increased TAG but decreased MGDG content [27]. Consequently, it was speculated that the mechanism of induction of lipid remodeling by inhibition of MGDG synthesis was involved in high activation of the key enzymes related to lipid metabolism and increased accumulation of ROS due to photo-oxidative damage under high light energy. In addition, ER stress might simultaneously contribute to accelerated TAG accumulation. A hypothetical lipid synthesis pathway of *Cr-mgd1* is briefly shown in Fig. 5.

### The improvement of lipid productivity in *Cr-mgd1* cultured under high light conditions

Light intensity is closely related to the growth and metabolism of photosynthetic organisms. While appropriate light intensity can increase the microalgal growth, lipid production, and pigment content, excess light energy beyond the threshold of photoinhibition can cause biological damage, leading to impaired biomass production. However, acclimation response to excess light could be applied as one of the strategies for increasing metabolic activity to modulate the quality of biomass and increase biomass productivity [28]. When *C. reinhardtii* and *Phaeodactylum tricornutum* were cultured under 500 µmol/m^2^/s and 300 µmol/m^2^/s, respectively, biomass productivity and TAG content were increased compared with cells cultured under 50 µmol/m^2^/s [29, 30]. The results of optimization of the biomass and lipid productivity in *Chlorococcum oleofaciens* under different light intensities proved that light intensity is important to enhance biomass properties, and influences the composition of metabolites and helps in achieving maximum productivity [31].

**Fig. 6:**
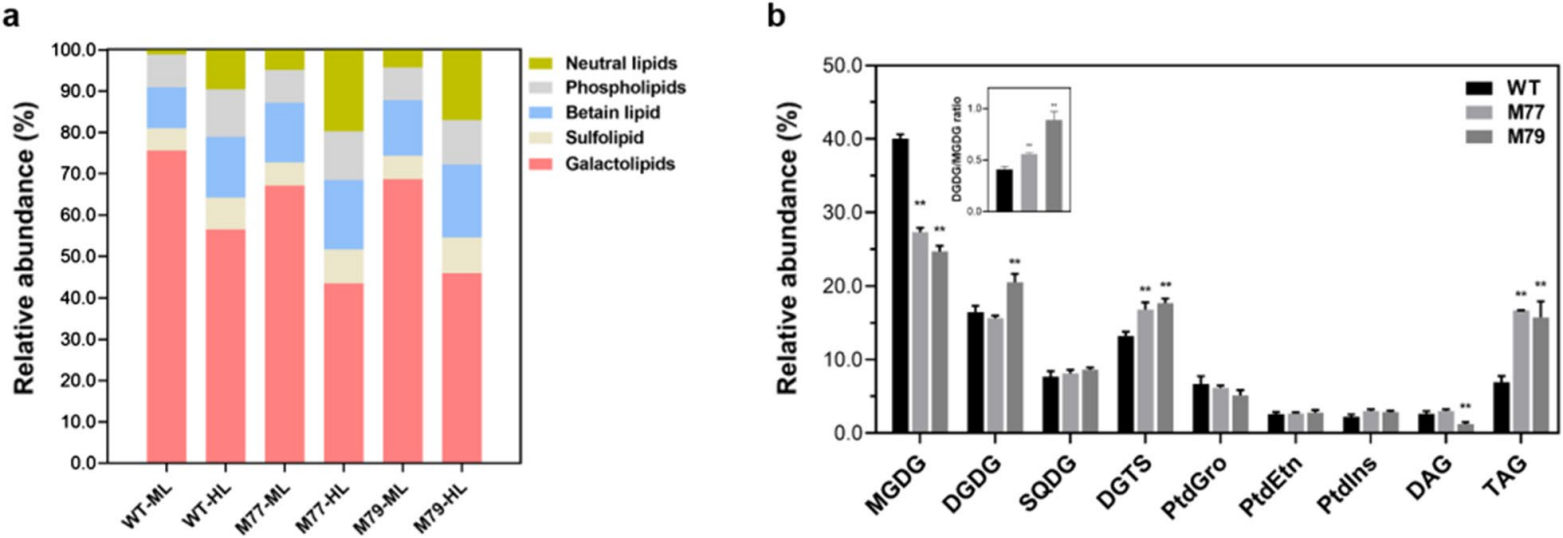
Change of lipid classes under high light conditions in the WT and *Cr-mgd1.* **a** Total lipid classes composition. **b** Quantitative analysis of membrane lipids. Profiling of lipids was performed using GC–FID and was normalized to dry cell weight. Significant differences between WT and *Cr-mgd1* are represented with asterisks based on Holm–Sidak method (**P < 0.01), and the error bars represent the standard deviation (SD). WT: wild-type; MGDG: monogalactosyldiacylglycerol; DGDG: digalactosyldiacylglycerol; SQDG: sulfoquinovosyldiacylglycerol; DGTS: digalactosyl-*N*,*N*,*N*-trimethylhomoserine; PtdGro: phosphatidylglycerol; PtdEtn: phosphatidylethanolamine; PtdIns: phosphatidylinositol; DAG: diacylglycerol; TAG: triacylglycerol

To increase neutral lipid productivity of *Cr-mgd1*, the lipid profile of the cells was investigated that were cultivated under high light (HL; 400 µmol photons/m^2^/s), which was determined to be the saturation light intensity based on pulse amplitude modulation (PAM) analysis. HL affected lipid remodeling in both WT and *Cr-mgd1* similarly and resulted in the decrease in galactolipids but increase in betaine lipid and neutral lipids (Fig. 6a); a high decrease in galactolipids and a great increase in neutral lipids were observed. In *Cr-mgd1,* under HL condition (*Cr-mgd1*–HL), the decrease in galactolipid content below 44% was related to the high accumulation of neutral lipids (up to 11%).

**Fig. 7:**
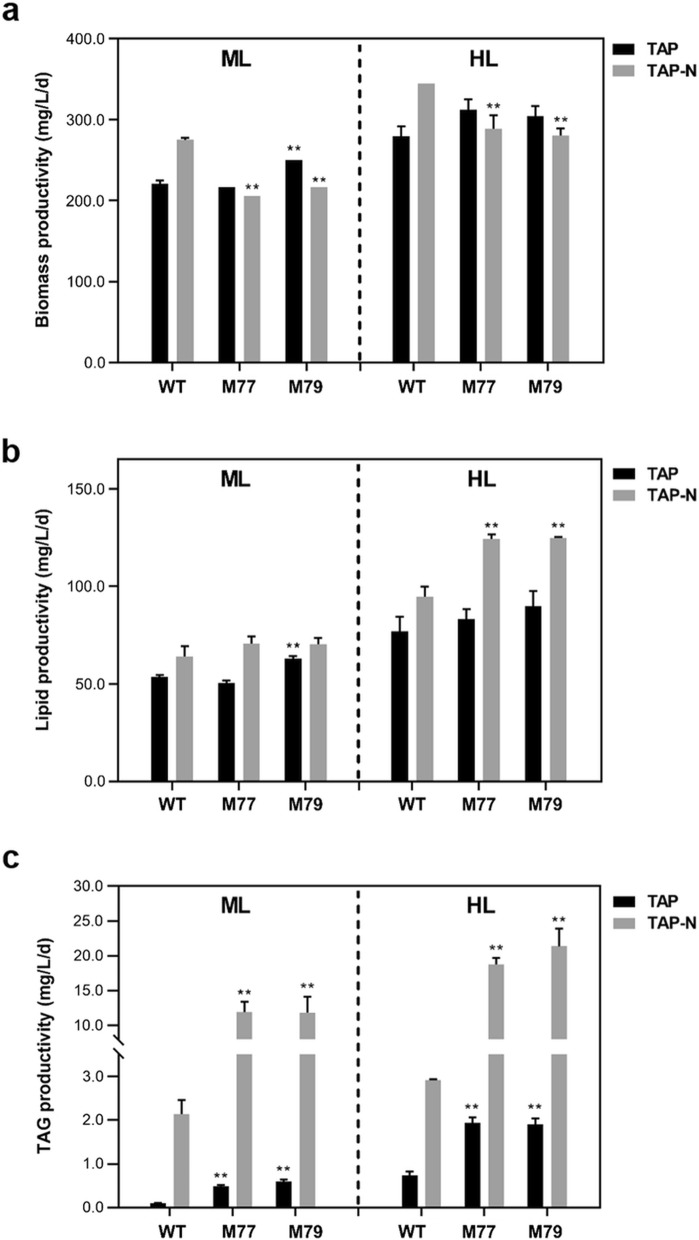
Comparison of biomass, lipid, and TAG productivity in each culture condition. **a** Biomass productivity. **b** Lipid productivity. **c** TAG productivity. Significant differences between WT and *Cr-mgd1* are represented with asterisks based on Holm–Sidak method (**P < 0.01), and the error bars represent the standard deviation (SD). WT: wild-type; TAP-N: nitrogen starvation TAP medium; ML: moderate light (120 µmol photons/m^2^/s); HL, high-light (400 µmol photons/m^2^/s)

Membrane lipid profiling revealed that the MGDG content of *Cr-mgd1–HL* was decreased by 33%, and TAG was increased 2.3-fold compared to WT–HL (Fig. 6b). Although the contents of DGDG and DAG in M77 and M79 were adversely changed, these did not seem to be associated mainly with the TAG content. While biomass productivity of *Cr-mgd1*–HL (308.33 mg/L/d) was significantly higher than that of WT–HL (269.72 mg/L/d) and *Cr-mgd1*–ML (moderate light; 233.33 mg/L/d)*,* total lipid content was not significantly different among them, and this resulted in no change in the lipid productivity under HL (Fig. 7a, b; Table 1). Conversely, TAG productivity was 1.99 mg/L/d in *Cr-mgd1*–HL, which was 2.71times more than that in WT–HL (Fig. 7c).

**Table 1:** Total lipid contents and TAG contents under each cultivation condition

Cell lines	ML		HL		ML–NS		HL–NS	
	Total lipid contents (%)	TAG conc (µg/mL)	Total lipid contents (%)	TAG conc (µg/mL)	Total lipid contents (%)	TAG conc (µg/mL)	Total lipid contents (%)	TAG conc (µg/mL)
WT	24.25 ± 0.50	0.40 ± 0.01	22.92 ± 0.88	2.93 ± 0.36	28.00 ± 3.00	12.81 ± 1.95	27.50 ± 1.50	17.46 ± 0.14
M77	23.33 ± 0.58	1.92 ± 0.14	22.68 ± 2.08	7.74 ± 0.51	40.50 ± 2.50	73.57 ± 2.26	43.00 ± 1.00	115.22 ± 2.81
M79	25.17 ± 0.58	2.37 ± 0.19	23.17 ± 0.76	7.60 ± 0.55	41.50 ± 3.50	81.23 ± 3.56	44.50 ± 1.50	139.62 ± 3.83

Data are mean ± standard deviation (SD) of triplicates. ML: moderate light (120 µmol photons/m^2^/s); HL: high-light (400 µmol photons/m^2^/s); ML–NS: moderate light with nitrogen starvation; HL–NS: high light with nitrogen starvation

Therefore, these results imply that excess light plays a critical role not only in inducing lipid remodeling and accumulation of neutral lipids but also in increasing the biomass. In addition, *Cr-mgd1* was improved as the enhancement of TAG synthesis under 400 µmol/m^2^/s light condition was particularly higher than that in WT–HL and WT–ML due to lipid remodeling by inhibition of MGDG synthesis. To achieve maximum lipid content and increase the amount of neutral lipids, nitrogen starvation stress was applied to *Cr-mgd1* with HL.

### Triacylglycerol overproduction strategy in *Cr-mgd1*

*Cr-mgd1* was cultured in nitrogen sufficient conditions under ML and HL for 4 d, and then each culture was transferred to nitrogen starvation condition (NS) maintained under same light conditions for 2 d. Regardless of the light intensity, NS increased the total lipid content and TAG content in WT and *Cr-mgd1.* Interestingly, the increment in *Cr-mgd1* was higher than that in WT. While the lipid content of WT–NS was 28% of dry cell weight (DCW), the lipid content of *Cr-mgd1–NS* was increased to up to 44.5% of DCW. TAG content in *Cr-mgd1*–HL–NS was the highest (120.21 mg/L), and it was 6.9-fold higher than that in WT–HL–NS (17.46 mg/L). Lipid and TAG productivity of *Cr-mgd1*–HL–NS (124.55 mg/L/d and 20.03 mg/L/d, respectively) were higher than those of *Cr-mgd1*–ML–NS (86.58 mg/L/d and 12.27 mg/L/d, respectively) (Fig. 7b, c), and these were increased by 31% and sevenfold, respectively, compared to WT–HL–NS (94.72 mg/L/d and 2.91 mg/L/d, respectively).

In ML– and HL–NS conditions, total fatty acid composition of *Cr-mgd1* exhibited increased SFAs and MUFAs but decreased polyunsaturated fatty acids (PUFAs) (Table 2). These changes in the degree of fatty acid saturation in *Cr-mgd1* improved the biomass properties for biodiesel production. In *Cr-mgd1*–HL–NS, the biomass properties were most suitable for the following three factors of biodiesel quality, the cetane number (CN), iodine value (IV), and oxidation stability (Table 2).

**Table 2:** Total fatty acid composition in wild-type (WT) and *Cr-mgd1* under nitrogen starvation

Fatty acid (% weight)	ML–NS			HL–NS		
	WT	M77	M79	WT	M77	M79
SFAs	30.90 ± 0.47	33.18 ± 0.19	32.41 ± 0.79	33.09 ± 0.17	36.82 ± 0.02	36.29 ± 0.18
MUFAs	11.14 ± 0.19	12.22 ± 0.42	13.72 ± 0.25	15.29 ± 0.21	21.34 ± 0.96	23.74 ± 1.47
PUFAs	61.53 ± .029	58.10 ± 0.80	57.65 ± 1.08	54.14 ± 0.39	43.10 ± 0.99	41.24 ± 1.33
Biodiesel properties^a^						
CN (≥ 47)	35.41	38.66	38.48	40.27	46.88	47.45
IV (≤ 120)	169.35	155.12	155.9	148.33	119.24	116.76
Oxidation stability (≥ 6 h)	5.56	5.64	5.68	5.81	6.40	6.56

^a^Biodiesel specifications from ASTM D6751 and EN 14214. Data represent mean ± standard deviation (SD) of triplicates. SFAs: saturated fatty acids; MUFAs: monounsaturated fatty acids; PUFAs: polyunsaturated fatty acids; CN: cetane number; IV: iodine value; ML–NS: moderate light with nitrogen starvation; HL–NS: high light with nitrogen starvation

Although the lipid content could be further increased depending on the cultivation period and the type of nutrient starvation, the short cultivation period is preferred to achieve the high biomass and lipid productivity. In *Chlamydomnas*, the average lipid content (% DCW) is estimated to be approximately 20% under normal conditions and is increased up to ~ 50% under stress conditions depending on the strain’s properties, such as cell-wall less, starch-less, and biosynthesis gene over-expressed/knock-out mutants [32–34]. The strain *BAFJ5*, which is one of the mutants defective in the small subunit of ADP–glucose pyrophosphorylase, showed increase in the total lipid content by approximately 46.4% of DCW under high-light and N starvation condition by reallocating the photosynthetically assimilated carbon from starch synthesis to neutral lipid synthesis [35]. However, growth inhibition was observed in *BAFJ5,* and a final lipid productivity of 95 mg/L/d was achieved. In comparison, *Cr-mgd1,* which originated from wild type, did not show impairment of biosynthesis and the cell growth was improved under high-light condition. The lipid productivity of *Cr-mgd1* was increased by 31% (124.55 mg/L/d), compared to *BAFJ5* under high-light and N starvation. Maximum neutral lipid productivity of 56.13 mg/L/d was achieved in *Cr-mgd1* during the 2-day cultivation under HL–NS condition. Neutral lipids are mainly increased in the form of TAGs through the induction of lipid synthesis under N starvation. For biofuel production, neutral lipids are more preferred than glycolipids or phospholipids based on their properties, such as higher percentage of fatty acids, possibility of induced high accumulation, and lack of phosphorous and sulfur [1]. Moreover, the demand for algal neutral lipids is recently increasing in cosmeceutical and food industries as a replacement of extracted plants due to their high productivity. These are usually used in cosmetic products as fragrance carriers, neutral bases, and bioactive ingredients for their moisturizing and softening properties, and for providing texture [36]. *Chlamydomonas* was certified as ‘Generally Recognized As Safe’ (GRAS), and its pharmaceutical industrial application is increasing in the global market. Thus, their improved properties with high lipid productivity and neutral lipid productivity in *Cr-mgd1* could be critical for their successful application in biofuel, food, and cosmeceutical industries.

Furthermore, lipid remodeling by regulation of membrane synthesis-related genes could be applied in oleaginous microalgae as well as starch-less strains of *Chlamydomonas*. Finally, a synergic effect is expected on the high accumulation of lipid content and successful achievement of the highest lipid productivity with optimized cultivation.

## Conclusions

The genetic regulation of lipid metabolism in microalgae has been widely attempted to increase the lipid productivity to develop biodiesel feedstock. Induction of membrane lipid remodeling was also involved in controlling the target lipid production. In this study, membrane lipid remodeling was induced in *Cr-mgd1* that led to changes in the lipid composition, induction of ER stress, and ROS accumulation. All these changes contributed to the improvement of the lipid properties for biodiesel feedstock as well as TAG accumulation. Therefore, the genetic regulation of MGDG synthesis could be an improvement strategy for microalgae biodiesel production.

## Methods

### Strain and cultivation

The *Chlamydomonas reinhardtii* CC-124 was obtained from the *Chlamydomonas* Resource Center (University of Minnesota, USA). *C. reinhardtii* was cultivated in a tris–acetate–phosphate (TAP) medium under continuous exposure to ML (120 µmol photons/m^2^/s) at 25 ℃ ± 0.5 in a shaking incubator. All experiments were performed in the TAP medium until stationary phase. Cells from the stationary phase were transferred to the TAP medium without a nitrogen source (TAP-N) and incubated for 2 d. *C. reinhardtii* was cultivated in the TAP medium under continuous light (400 µmol photons/m^2^/s) at 25 ℃ ± 0.5 in a shaking incubator for high light stress condition. All cultivations and experiments were performed in triplicates.

### Vector construction for *CrMGD1* knockdown and transformation

The *MGD1* (Cre13.g585301.t1.1) was obtained from the Phytozome database (http://phytozome.net). The pChlamiRNA3-int (obtained from the Chlamydomonas Resource Center) was treated with SpeI restriction enzyme (Enzynomics, Korea), and *MGD1* amiRNA was replaced with creMIR1157 [37]. To knockdown the *MGD1* gene, the 21-nucleotide sequence of amiRNA (ACGCTCTTACCCAACGAGAGC) was designed with two mismatched bases at the 4th and 21st positions from the 5′ UTR of *CrMGD1* gene. The vector generated through cloning was named pCrMGD–RNAi and amplified with F-pCre–RNAi-mgd and R-pCre–RNAi-mgd primers to confirm the sequence. *Chlamydomonas* cells were harvested at 3 × 10^6^ cells/mL and were washed twice with MAX efficiency transformation reagent (Invitrogen, USA) and suspended in the same reagent at a final concentration of 1 × 10^7^ cells/mL. A total of 1 µg of the linearized pCrMGD–RNAi vector was incubated with the protoplasts at 4 ℃ for 10 min. Electroporation was performed using a gene pulser (Bio-Rad, USA) at 750 voltage (V), 25 microfarad (μF), and 20 ohms (Ω) of resistance by exponential decay wave. The transformed cells were recovered by incubation for 16 h in TAP medium containing 60 mM sucrose. Thereafter, the cells were spread on TAP agar medium containing 5 µg/mL paromomycin. One week later, colony PCR was performed using the *aphVIII* primers. Colonies with amplified *aphVIII* region were transferred to liquid TAP medium containing 10 µg/mL paromomycin.

### Southern blot analysis

The DNA was extracted using phenol:chloroform:isoamyl alcohol solution (25:24:1, Sigma Aldrich, USA). A total of 15 µg of the extracted DNA was fragmented by incubation with the restriction enzyme *Pst*I (Enzynomics, Korea) at 30 °C for 16 h and separated by electrophoresis on 0.8% agarose gel. The separated DNA was transferred to a Hybond N + membrane (Amersham Biosciences, Sweden) by capillary transfer. The probe used for detection was *aphVIII *(200 bp) and labeled with ^32^P. Hybridization was performed in a hybridization chamber at 65 °C for 16 h. The membrane was washed twice with 2 × saline sodium citrate (SSC) and 0.5 × SSC, and ^32^P was detected using a bio-imaging analyzer system (Fujifilm, Japan).

### Quantitative real-time PCR (qRT-PCR) analysis for gene expression

RNA was isolated using Trizol (Ambion, USA) and QIAGEN RNeasy kit (Qiagen, USA), and the contaminated genomic DNA (gDNA) was removed using RQ1 RNase-Free DNase kit (Promega, USA). Total RNA (500 ng) was used to synthesize cDNA using GoScript™ Reverse Transcription System (Promega, USA). The relative expression levels were calculated with the 2^−ΔΔCT^ method using *Chlamydmonas* β-subunit-like polypeptide (*CBLP*) as the housekeeping gene. qPCR was performed with a CFX Connect Real-Time System (BioRad, USA) under the following reaction conditions: 95 °C for 15 min followed by 44 cycles of 95 °C for 20 s, 60 °C for 30 s, and 72 °C for 20 s, and final extension at 72 °C for 1 min. The sequences of all primers are listed in Additional file 6.

### Measurement of photosynthetic activities

The cells were cultivated in TAP medium under continuous light (120 µmol photons/m^2^/s) at 25 ℃ for 4 d. Cells were then acclimated to the dark for 10 min prior to the measurement of photosynthetic parameters. Effective photochemical quantum yields of PSII (Y(II)) and rETR were measured using “Light curve” program in a mini-pulse amplitude modulation (mini-PAM II, Heinz Walz, Germany). rETR was calculated as: yield × photosynthetically active radiation (PAR) × 0.5 × absorptivity (0.84). The “Light curve” program measured different actinic lights from 0 to 1500 µmol photons/m^2^/s. NPQ was measured using the “Actinic + recovery” program under 1500 µmol photons/m^2^/s actinic light for 14 min at 30 s intervals.

### Total lipid extraction and quantification

The cells cultivated in TAP medium and TAP-N were harvested and subjected to freeze-drying. The lipids were extracted using 10 mg of dried cells as described previously [27]. The lipid phase was transferred to an aluminum dish and evaporated in a fume hood overnight. Dried lipids were eluted with chloroform to a final con80/30/1 (v/v/v) centration of 10 mg/mL. Thin-layer chromatography (TLC) was performed for lipid class separation on a silica gel plate (MSD, USA). To separate polar lipids, silica plate was developed on methyl acetate/isopropanol/chloroform/methanol/0.25% KCl (25/25/25/10/4, v/v/v/v/v) solvent mixture. Neutral lipids were separated by hexane/diethyl ether/acetic acid (80/30/1, v/v/v) solvent mixture. The developed silica plate was stained with 0.01% (w/v) primuline (Sigma Aldrich, USA) dissolved in 80% acetone. Detected lipid bands were isolated from the plate and converted to fatty acid methyl esters via an acid-catalyzed transesterification method. Gas chromatography (GC) (SHIMADZU, GC-2010, Japan) was used for the quantitative analysis of lipids.

### Determination of ROS content using dichlorofluorescein diacetate (DCFH-DA) assay

To determine ROS content in the cell, 1 mL of cell culture was harvested and washed twice with phosphate buffer (pH 7.0). The washed cells were suspended in 1 mL phosphate buffer and treated with DCFH-DA (Sigma-Aldrich, UK), followed by incubation for 30 min in the dark via shaking at 30 rpm. Thereafter, intensity of fluorescence was measured using a fluorescence microplate reader. The excitation and emission wavelengths were 485 and 535 nm, respectively.

### Statistical analysis

All experiments were performed in triplicates. The results are shown as mean ± standard deviation (SD), and the error bars represent the SD. Asterisks indicate significant differences between WT and *Cr-mgd1* based on Holm–Sidak method (**P < 0.01, *P < 0.05).

## Supplementary Information

**Additional file 1.** Southern blotting for confirmation of integrated pCrMGD1 vectors into *Chlamydomonas* gDNA.

**Additional file 2.** Relative expression of fatty acids desaturases in *Chlamydomonas* cells.

**Additional file 3.** Total fatty acid composition in *Cr-mgd1* mutant.

**Additional file 4.** The concentration of DAG (a) and free fatty acids (b) under moderate light and high-light conditions.

**Additional file 5.** The ROS level of *Cr-mgd1* mutant.

**Additional file 6.** Primer list in this study.

## Data Availability

All data generated or analyzed during this study are included in this published article and Additional file 1.

## Abbreviations

MGDG: Monogalactosyldiacylglycerol
MGD1: MGDG synthase 1
SFAs: Saturated fatty acids
MUFAs: Monounsaturated fatty acids
PUFAs: Polyunsaturated fatty acids
qRT-PCR: Quantitative real-time PCR
TAG: Triacylglycerol
DGTS: Diacylglyceryltrimethylhomoserines
CrΔ4FAD: MGDG-specific Δ4-desaturase from *Chlamydomonas*
CrFAD6: Fatty acid desaturase 6 from *Chlamydomonas*
DGAT1: Diacylglycerol *O*-acyltransferase1
PDAT: Phospholipid:diacylglycerol acyltransferase
LCS2: Long-chain acyl-CoA synthetase2
MLDP: Major lipid droplet protein
PGD1: Plastid galactiglycerolipid degradation1
SOD: Superoxide dismutase
CAT1: Catalase 1
APX1: Ascorbate peroxidase1
GPX5: Glutathione peroxidase5
GPAT1: Glycerol 3-phosphate
CrLPAAT2: 2‐Lysophosphatidic acid acyltransferase 2 from *Chlamydomonas reinhardtii*
PAP2: Phosphatidic acid phosphatase 2
BiP1: Binding protein 1
CAL2: Calreticulin 2
CrIRE: Inositol-requiring enzyme 1 from *Chlamydomonas reinhardtii*
TLC: Thin layer chromatography
