# Supplementary material for: Inhibition of monogalactosyldiacylglycerol synthesis by down-regulation of MGD1 leads to membrane lipid remodeling and enhanced triacylglycerol biosynthesis in Chlamydomonas reinhardtii
Source: Biotechnol Biofuels Bioprod. 2022 Aug 27;15:88. doi: 10.1186/s13068-022-02187-x (PMC9419350; doi:10.1186/s13068-022-02187-x)
Supplement: Supplementary file 2 — Additional file 2. Relative expression of fatty acids desaturases in Chlamydomonas cells. [file 13068_2022_2187_MOESM2_ESM.docx]

**Inhibition of monogalactosyldiacylglycerol synthesis by down-regulation of MGD1 leads to membrane lipid remodeling and enhanced triacylglycerol biosynthesis in *Chlamydomonas reinhardtii***

Jun-Woo Lee ^a,b,c^, Min-Woo Lee ^a,d^, Chun-Zhi Jin ^a^, Hee-Mock Oh ^a,d^, EonSeon Jin ^b^, and Hyung-Gwan Lee ^a,d*^

^a^ Cell Factory Research Center, Korea Research Institute of Bioscience and Biotechnology (KRIBB), Daejeon 34141, Republic of Korea

^b^ Department of Life Science, Hanyang University, Seoul 04763, Republic of Korea

^c^ LMO Team, National Institute of Ecology, Seocheon-gun 33657, Republic of Korea

^d^ Department of Environmental Biotechnology, University of Science & Technology (UST), Daejeon 34113, Republic of Korea

*Corresponding author

Dr. Hyung-Gwan Lee

trustin@kribb.re.kr


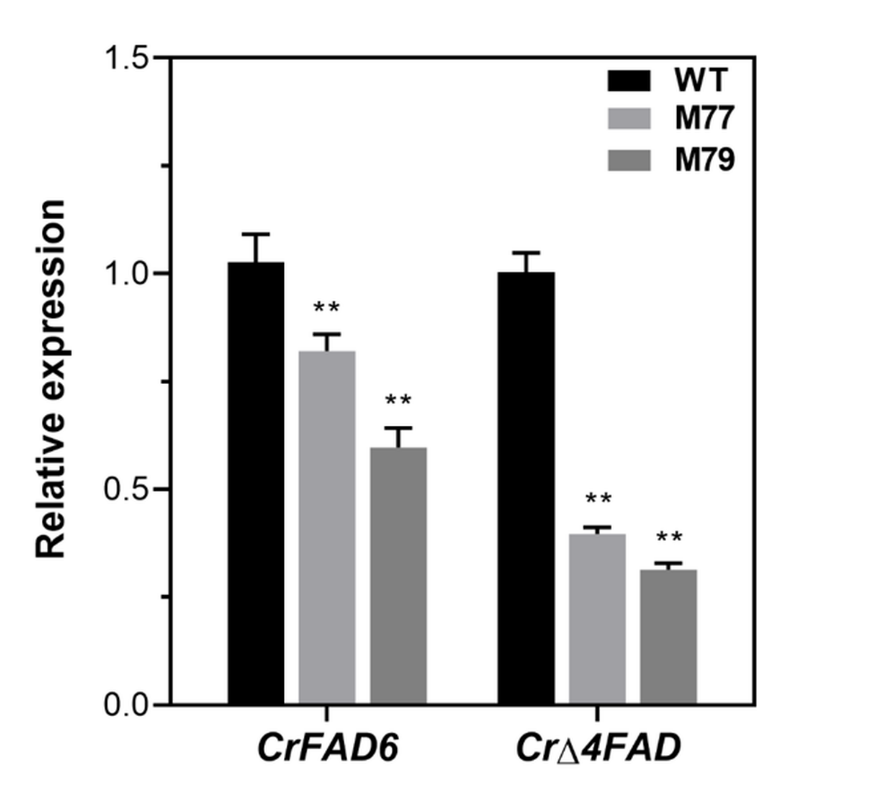


**Additional file 2.** Relative expression of fatty acids desaturases in *Chlamydomonas* cells.

Relative expression levels were calculated by normalizing the values to those of *Chlamydmonas* β-subunit-like polypeptide (CBLP). All experiments were performed in biological triplicates. Significant differences between WT and *Cr-mgd1* are represented with asterisks based on Holm-Sidak method (**P < 0.01). The error bars represent the standard deviation (SD). CrFAD6, ω-6-fatty acid desaturase from *Chlamydomonas* *reinhardtii*; CrΔ4FAD, MGDG-specific Δ-4 fatty acid desaturase from *Chlamydomonas* *reinhardtii*.
