# Supplementary material for: Inhibition of monogalactosyldiacylglycerol synthesis by down-regulation of MGD1 leads to membrane lipid remodeling and enhanced triacylglycerol biosynthesis in Chlamydomonas reinhardtii
Source: Biotechnol Biofuels Bioprod. 2022 Aug 27;15:88. doi: 10.1186/s13068-022-02187-x (PMC9419350; doi:10.1186/s13068-022-02187-x)
Supplement: Supplementary file 5 — Additional file 5. The ROS level of Cr-mgd1 mutant. [file 13068_2022_2187_MOESM5_ESM.docx]

**Inhibition of monogalactosyldiacylglycerol synthesis by down-regulation of MGD1 leads to membrane lipid remodeling and enhanced triacylglycerol biosynthesis in *Chlamydomonas reinhardtii***

Jun-Woo Lee ^a,b,c^, Min-Woo Lee ^a,d^, Chun-Zhi Jin ^a^, Hee-Mock Oh ^a,d^, EonSeon Jin ^b^, and Hyung-Gwan Lee ^a,d*^

^a^ Cell Factory Research Center, Korea Research Institute of Bioscience and Biotechnology (KRIBB), Daejeon 34141, Republic of Korea

^b^ Department of Life Science, Hanyang University, Seoul 04763, Republic of Korea

^c^ LMO Team, National Institute of Ecology, Seocheon-gun 33657, Republic of Korea

^d^ Department of Environmental Biotechnology, University of Science & Technology (UST), Daejeon 34113, Republic of Korea

*Corresponding author

Dr. Hyung-Gwan Lee

trustin@kribb.re.kr


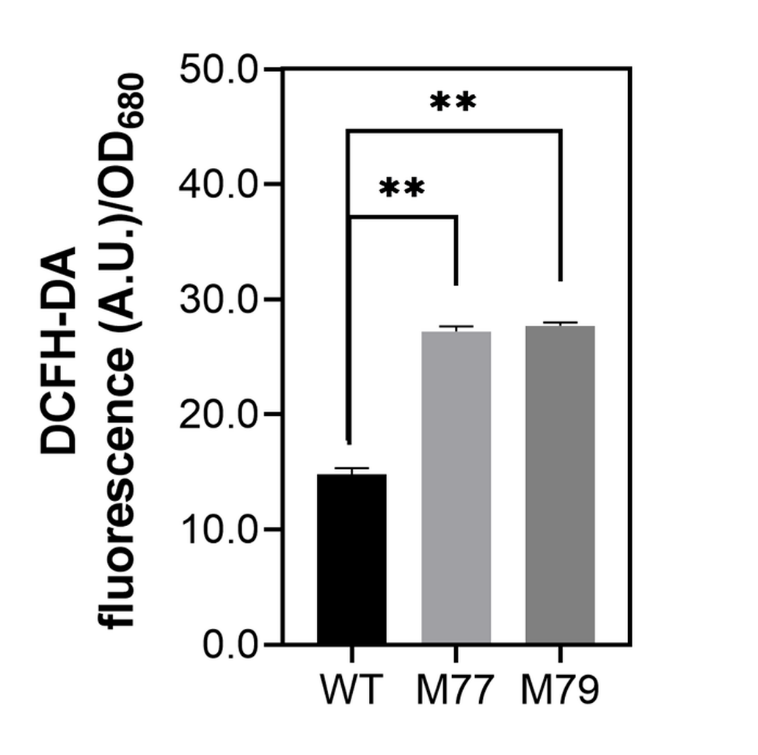


**Additional file 5.** Measurement of cellular ROS level using dichlorofluorescin diacetate (DCFH-DA) staining.

Relative DCFH-DA fluorescence normalized to optical density (OD680) of *C. reinhardtii* cells. Significant differences between WT and *Cr-mgd1* are represented with asterisks based on Holm-Sidak method (**P < 0.01), and the error bars represent the standard deviation (SD). WT, wild-type**.**
