# Supplementary material for: Inhibition of monogalactosyldiacylglycerol synthesis by down-regulation of MGD1 leads to membrane lipid remodeling and enhanced triacylglycerol biosynthesis in Chlamydomonas reinhardtii
Source: Biotechnol Biofuels Bioprod. 2022 Aug 27;15:88. doi: 10.1186/s13068-022-02187-x (PMC9419350; doi:10.1186/s13068-022-02187-x)
Supplement: Supplementary file 6 — Additional file 6. Primer list in this study. [file 13068_2022_2187_MOESM6_ESM.docx]

**Inhibition of monogalactosyldiacylglycerol synthesis by down-regulation of MGD1 leads to membrane lipid remodeling and enhanced triacylglycerol biosynthesis in *Chlamydomonas reinhardtii***

Jun-Woo Lee ^a,b,c^, Min-Woo Lee ^a,d^, Chun-Zhi Jin ^a^, Hee-Mock Oh ^a,d^, EonSeon Jin ^b^, and Hyung-Gwan Lee ^a,d*^

^a^ Cell Factory Research Center, Korea Research Institute of Bioscience and Biotechnology (KRIBB), Daejeon 34141, Republic of Korea

^b^ Department of Life Science, Hanyang University, Seoul 04763, Republic of Korea

^c^ LMO Team, National Institute of Ecology, Seocheon-gun 33657, Republic of Korea

^d^ Department of Environmental Biotechnology, University of Science & Technology (UST), Daejeon 34113, Republic of Korea

*Corresponding author

Dr. Hyung-Gwan Lee

trustin@kribb.re.kr

**Additional file 6.** Primer list in this study

| Primer name | Sequence | Usage | Reference |
| --- | --- | --- | --- |
| F-pCre-RNAi-mgd | CGGAATTCGGTGTTGGGTCGGTGTTTTTG | Confirmation of pCrMGD1 insertion | In this study |
| R-pCre-RNAi-mgd | CGGAATTCCGGTGTAACTAAGCCAGCCCA |  |  |
| F-aphVIII | ATTCCCGTACCTCGTGTTGT | Southern blotting | Lee et al. 2017 |
| R-aphVIII | CTCGTCCAGATCCTCCAAGT |  |  |
| F-MGD1 | cgcACGCTCTTACCCAACGAGAG | qRT-PCR | In this study |
| R-MGD1 | cttGTTGACATTGCCAGAAGTGCt |  |  |
| F-SOD | GGCTGTCAGTTTTGTTTCCCTCGT | qRT-PCR | In this study |
| R-SOD | GACTTTCATGTTCCTCGCCAGCT |  |  |
| F-CAT1 | CAGGAGGCTGCAGGAAAACTGAT | qRT-PCR | In this study |
| R-CAT1 | TACATGACAACATGTACATTACGCA |  |  |
| F-APX1 | AAGGCGGAGCTCTACAACTACAT | qRT-PCR | In this study |
| R-APX1 | GGTACTTCTTTTTGATGGGGTTC |  |  |
| F-GPX5 | GGAGCTGTACCAACAGTACAAGG | qRT-PCR | In this study |
| R-GPX5 | CTCCATCATGAACTGCTTCTTTT |  |  |
| F-FAD6 | TTGCGCGGGCGGCTTTGTTC | qRT-PCR | Anja Hemschemeier et al. 2013 |
| R-FAD6 | CACCGCTGCTCCACAGACAC |  |  |
| F-CrΔ4FAD | CCGCAATCTCCTTCATGCACTA | qRT-PCR | In this study |
| R-CrΔ4FAD | TAGAACTTGGAGAGCATCAGCAT |  |  |
| F-BiP1 | AGTGAGCCCGTCTTTTAGAACTT | qRT-PCR | Lee et al. 2017 |
| R-BiP1 | TCTCCTCTGTACCACCGTTTTTA |  |  |
| F-CAL2 | TGACTACGTCCACGACGACAAG | qRT-PCR | In this study |
| R-CAL2 | CCAGGTGTCCTCAGCGAACTTC |  |  |
| F-CrIRE | CTTGGCAGTTTGCCAGTACATT | qRT-PCR | In this study |
| R-CrIRE | AGTCCAGGTAGGACCCGAGTCA |  |  |
| F-GPAT | CAACACTCTCATCCTCTTGTTCC | qRT-PCR | In this study |
| R-GPAT | TACACAGCACCACGTAGTCAATC |  |  |
| F-CrLPAAT2 | ATTCTTGTTCTCCGTGTTTGTGT | qRT-PCR | In this study |
| R-CrLPAAT2 | GAGGTAGGCGTCAATGAAGAAGT |  |  |
| F-PAP2 | GCGTGTTTGCCTACTTCCTC | qRT-PCR | In this study |
| R-PAP2 | CACTACTCGCGCCGTACAT |  |  |
| F-DGAT1 | CCCCAAGCAGACGGCAAACAAT | qRT-PCR | In this study |
| R-DGAT1 | CACACGGTCACATCAGCAAACT |  |  |
| F-PGD1 | GTCATTGCCTTGTTGATTGGTAT | qRT-PCR | In this study |
| R-PGD1 | ACATGGCTAATAGAAGAGGCACA |  |  |
| F-PDAT | TTGCTATCGTTTTGGGTTCTTGA | qRT-PCR | In this study |
| R-PDAT | GCCTGACGACATCACCTAAGA |  |  |
| F-LCS2 | CGTTTGATCGAGACTCCTGAA | qRT-PCR | In this study |
| R-LCS2 | CAGCTATCGCCCATCTCATAC |  |  |
| F-CBLP | GACGACCTGCGCCCCGAGTT | qRT-PCR | Lee et al. 2017 |
| R-CBLP | AGGCGCGGCTGGGCATTTAC |  |  |
